# Cancer's new Achilles' heel?

**DOI:** 10.18632/oncotarget.19954

**Published:** 2017-08-05

**Authors:** Gareth I. Owen

**Affiliations:** Faculty of Biological Sciences & Faculty of Medicine, Pontificia Universidad Católica de Chile, Santiago, Chile

**Keywords:** ASncmtRNA, mitochondrial non-coding RNA, oligonucleotide

**Figure 1:**
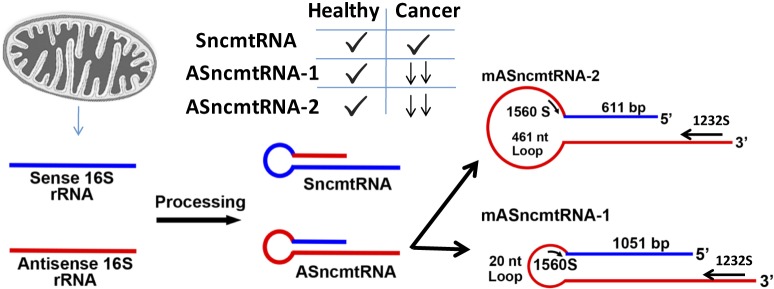
Derivation of sense and anti-sense non-coding mitochondrial RNAs (modified images from references 1 and 2)

Claims to the discovery of cancers Achilles’ heel are multiple, even clichéd. Analogies to this Greek myth usually refer to the fall of the undefeatable, yet the novelty behind the legend is that a seemingly immortal foe could be toppled by something so innocuous as his heel. This may be the case for cancer and a non-coding mitochondrial RNA that as yet has no known physiological function or mechanism of action. Two papers presented in Oncotarget in 2016 and 2017 demonstrate that an oligonucleotide against a non-coding mitochondrial RNA can bring about tumor regression and prevent metastasis in pre-clinical cancer models [1, 2]. Originally identified in the sperm nucleus [3], the team of researchers led by Luis Burzio and Veronica Burzio (father & daughter) introduced an unexpected connection between cancer and non-coding mitochondrial transcripts that are derived from the differential splicing and ligation of 16S sense and antisense rRNA in their seminal study published in 2009 [4]. Although they are not perfectly-matched sense/anti-sense, the resultant stem-loop structures display different patterns of expression in healthy versus malignant cells (Figure 1). Healthy cells normally express two anti-sense mitochondrial transcripts along with a sense transcript that is present in proliferating cells. This sense transcript is also present in malignant cells, however anti-sense mitochondrial transcripts are severely down-regulated, being almost undetectable. While the authors have demonstrated a differential expression of these anti-sense transcripts (called ASncmtRNA-1 and ASncmtRNA-2) that can distinguish between healthy and malignant cells, the Burzio team also discovered that when these anti-sense transcripts were further down-regulated, the cancer cells entered programmed cell death [4].

Derived from this observation, the Burzio laboratory immediately saw the potential for a cancer treatment. First they confirmed that these non-coding RNAs were also present in mice and proceeded to evaluate if a complementary (anti-sense) oligonucleotide to these anti-sense transcripts could indeed prevent or reduce the disease burden in preclinical models. Initially, two mouse anti-sense oligonucleotides with the ability to bind both anti-sense transcripts were designed and tested. As shown in Figure 1, a -3′ to -5′ oligonucleotide that binds the stem loop region of the mouse antisense 16S rRNA (ASO-1560S) and a second -5′ to -3′ oligonucleotide that binds the same antisense strand (ASO-1232S). These designed oligonucleotides and human oligosnucleotides were first tested for their ability to induce apoptosis in both mouse and human malignant cells [2, 3]. Then they demonstrated *in vivo* that this approach was effective in drastically reducing tumor burden and possibly eliminating the establishment of metastatic disease in preclinical models [1, 2]. Using a murine melanoma model, the Burzio laboratory recreated a clinical setting commonly seen in patients, waiting for a tumor to be established prior to resection and treatment. Thereby, C57BL/6 mice were subcutaneously injected with melanoma B16F10 cells, which resulted in measurable tumors after 12 days. Tumors were then surgically removed and animals received either a control oligonucleotide or ASO-1560S on alternate days by intravenous and intraperitoneal injections starting at day 3 post-surgery. In the control oligonucleotide injected animals, tumor progression was observed and animals were sacrificed at day 25 post-cancer cell injection. In sharp contrast, animals receiving ASO-1560S were still alive at day 120. Animals were then euthanized for examination. Remarkably, no signs of lung or liver metastases or relapse were observed in this group. Next, in a forced assay of metastasis, that injects cancer cells directly into circulation via the tail vein of animals, co-injection with ASO-1560S notably reduced lung metastasis. Demonstrating these results were not specific to this model, experiments were also replicated in a murine renal cell carcinoma model (RenCa) [2]. This time, RenCa cells were subcutaneously injected to establish a measurable tumor after 10 days. Mice were then intraperitoneally injected with control or ASO-1232S oligonucleotides. At day 26, control animals reached tumor burden limits defined by the ethical committee and were sacrificed. In ASO-1232S injected mice, tumor burden slowly increased over the first 10 days of treatment, then tumors started to shrink and eventually became undetectable. Mice were still healthy at day 150 post-cancer cell injections. Although the precise mechanisms of action of ASO-1232S and ASO-1560S are still largely unknown, both articles demonstrate a reduction in intratumoral levels of Survivin [1, 2]. The RenCa model also shows a drastic reduction in the levels of MMP9 [2]. Interestingly, the experimental approach to knockdown ASncmtRNA levels does not appear to modify its effect, as the use of a lentiviral-encoded shRNA against ASncmtRNAs also significantly retarded primary tumor growth and reduced lung metastasis in an *in vivo* model of melanoma [5]. While many published reports have shown promising preclinical results in a variety of specific models or specific tumor types, very few potential therapies demonstrate such consistency in terms of anti-tumoral activity across cell lines, cancer models and tumor types tested, as the strategy proposed by this group of investigators.

Apart from the potential development of treatments and diagnostics, the introduction to the scientific community of these non-coding mitochondrial RNAs may bring a new vision to cellular and cancer biology. Of course, many questions remain unanswered. What is (are) the cellular physiological function (s) of these transcripts? What is the mechanism of action in pathophysiology and why do non-cancerous cells not undergo apoptosis when the antisense transcripts are lost? Is the down-regulation of these non-coding mitochondrial RNAs an essential step in carcinogenesis? Interestingly, this group of investigators reported that strains of human papillomavirus (HPV), that are known to increase cervical cancer incidence, can down-regulate these transcripts. In 2015, a study by Bianchessi and colleagues reported that ASncmtRNA-2 (which is down-regulated in cancer) was up-regulated in aging and replicative senescence of human endothelial cells [6]. Moreover, they propose that two miRNAs, called hsa-miR-1973 and hsa-miR-4485, are derived from the ASncmtRNA-2 stem via Dicer-processing [6]. It is evident that this is a young, developing field with the potential to deliver long-overdue answers about non-coding RNA biology and the process of oncogenesis.

These findings published in Oncotarget are the solid proof-of-concept required in order to proceed with the oligonucleotide targeting of ASncmtRNA into clinical trials. The authors indicate that the United States Food and Drug Administration (FDA) has approved their Investigational New Drug Application for a human oligonucleotide and that a Phase I Clinical Trial is underway and close to completion in California, USA [7].

To date, ASncmtRNA therapy is “Cancer’s Achilles’ heel” only in mice, and therefore we wait, as does a child for Christmas, to find out whether this approach is applicable to cancer patients.
